# High frequency conductivity decomposition by solving physically constraint underdetermined inverse problem in human brain

**DOI:** 10.1038/s41598-023-30344-1

**Published:** 2023-02-25

**Authors:** Oh-In Kwon, Mun Bae Lee, Geon-Ho Jahng

**Affiliations:** 1 Department of Mathematics, College of Basic Science, Konkuk University, Seoul, 05029 Korea; 2 Department of Radiology, Kyung Hee University Hospital at Gangdong, College of Medicine, Kyung Hee University, Seoul, 05278 Korea

**Keywords:** Magnetic resonance imaging, Preclinical research

## Abstract

The developed magnetic resonance electrical properties tomography (MREPT) can visualize the internal conductivity distribution at Larmor frequency by measuring the B1 transceive phase data from magnetic resonance imaging (MRI). The recovered high-frequency conductivity (HFC) value is highly complex and heterogeneous in a macroscopic imaging voxel. Using high and low *b*-value diffusion weighted imaging (DWI) data, the multi-compartment spherical mean technique (MC-SMT) characterizes the water molecule movement within and between intra- and extra-neurite compartments by analyzing the microstructures and underlying architectural organization of brain tissues. The proposed method decomposes the recovered HFC into the conductivity values in the intra- and extra-neurite compartments via the recovered intra-neurite volume fraction (IVF) and the diffusion patterns using DWI data. As a form of decomposition of intra- and extra-neurite compartments, the problem to determine the intra- and extra-neurite conductivity values from the HFC is still an underdetermined inverse problem. To solve the underdetermined problem, we use the compartmentalized IVF as a criterion to decompose the electrical properties because the ion-concentration and mobility have different characteristics in the intra- and extra-neurite compartments. The proposed method determines a representative apparent intra- and extra-neurite conductivity values by changing the underdetermined equation for a voxel into an over-determined minimization problem over a local window consisting of surrounding voxels. To suppress the noise amplification and estimate a feasible conductivity, we define a diffusion pattern distance to weight the over-determined system in the local window. To quantify the proposed method, we conducted a simulation experiment. The simulation experiments show the relationships between the noise reduction and the spatial resolution depending on the designed local window sizes and diffusion pattern distance. Human brain experiments (five young healthy volunteers and a patient with brain tumor) were conducted to evaluate and validate the reliability of the proposed method. To quantitatively compare the results with previously developed methods, we analyzed the errors for reconstructed extra-neurite conductivity using existing methods and indirectly verified the feasibility of the proposed method.

## Introduction

The biological and electrical properties of tissues in the human brain are involved in various fields, such as acute or chronic pain controls, diagnoses of various diseases, analyses of brain functions, and clinical practices used to treat neuropathology. As an intrinsic biophysical property, the tomographic imaging of tissue’s electrical properties has been improved by recent developments in magnetic resonance (MR) imaging techniques. Various techniques have been developed and experimented to measure and analyze the electrical properties of biological tissues using a magnetic resonance imaging (MRI) scanner^1–7^. Magnetic Resonance Electrical Properties Tomography (MREPT) is one of the electrical property mapping techniques without applying any external electrical stimulation, but using a B1 mapping technique with the eddy currents induced by RF pulses. MREPT have successfully recovered the conductivity and permittivity distribution at Larmor frequency (about 128 MHz at 3T)^6,7^.

Recently, MREPT researches have been conducted to estimate the quantified conductivity values comparing to those reported in literature: tissue conductivity with and without boundary erosion^8^, investigated variation in reported human head electrical conductivity values^9,10^, and the effect of water content on electrical properties^11^. MREPT has been widely applied for numerous clinical studies : conductivity imaging for breast cancer detection^12,13^, conductivity and permittivity mapping of the in vivo rat brain with and without ischemia using ultra-high field (21.1 T)^14^, conductivity imaging for the assessment of glioblastomas using a rat brain tumor^15^, electrical properties for musculoskeletal tissues by evaluating the conductivity of muscle, cartilage, and peripheral nerve around the knee joint^16^, and conductivity characteristics for the brain of Alzheimer’s disease patients^17^.

An MREPT signal in a brain imaging voxel is contributed by a microstructure of a various range of sizes, shapes, composition, and electrical properties. In a macroscopic imaging voxel in the brain, typically, gray matter, white matter, and cerebral spinal fluid (CSF) can be heterogeneously mixed in the same voxel. The estimated conductivity value in a macroscopic voxel is highly complex and heterogeneous. In MREPT, the B1 phase signals include in the microscopic structure information of the intra- and extra-cellular induced by the secondary magnetic fields at Larmor frequency. To model B1 phase signal in MREPT in a microstructure level in a imaging voxel, we have to adapt a microstructural compartment model. To characterize and analyze a water molecule movement within and between compartments by inferring the microstructure and underlying architectural organization of tissues in the brain, tractable and feasible models have been proposed, such as composite hindered and restricted model of diffusion (CHARMED)^18^, neurite orientation dispersion and density imaging (NODDI)^19^, Bingham-NODDI^20^, and multi-compartment spherical mean technique (MC-SMT)^21,22^. MC-SMT was developed to evaluate microscopic features of the intra- and extra-neurite compartments in nervous tissue in the brain^21,22^ and overcomes several limitations of NODDI by avoiding the assumptions about the neurite orientation distribution (e.g. single orientations, spherical harmonics or mixtures of Bingham distributions). The MC-SMT model represents the diffusion signal as a weighted sum of restricted (intra-neurite) and hindered (extra-neurite) compartments, and estimates the separated microstructural diffusivity parameters. Since MC-SMT uses the spherical mean signal over the gradient directions for a specified *b*-value to detect microstructural parameters, MC-SMT shows feasible results with relatively small applied gradient directions, although more gradient directions are better to reflect the details of water molecules diffusivity. Recently, we proposed a new in vivo non-invasive imaging method using the MC-SMT model to extract the low-frequency dominant electrical properties from the high-frequency conductivity^23^. In this paper, we call the conductivity at Larmor frequency as high-frequency conductivity.

The high-frequency conductivity (HFC) of a macroscopic voxel can be represented as a decomposed form of the intra- and extra-neurite compartments with an appropriated microstructure model. Using the decomposed form, the problem to determine the intra- and extra-neurite conductivity from the HFC is an underdetermined linear inverse problem. In general, many combinations of intra-neurite conductivity and external neurite conductivity may produce the same HFC due to the lack of information. To overcome these difficulties, an electrodeless conductivity tensor imaging (CTI) was proposed using diffusion weighted image (DWI) data for multi-shell *b*-values^24^. However, the CTI method has to assume several strong constraints for implementation. Up to now, since there is no available way to estimate the ratio of ion concentrations in the intra- and extra-cellular spaces, the CTI method uses a fixed constant ratio of ion concentrations in the intra- and extra-cellular compartments. The presumed fixed ratio value, 0.41, has been typically used by adopting reference values of intra- and extra-cellular ion concentrations^2,23,24^. This strong assumption of the ratio of intra- and extra-cellular ion concentrations requires lots of theoretical and practical evidence to be accepted as an established MR imaging method to decompose the HFC.

A motivation of this paper is that the estimated intra-neurite volume fraction (IVF) can be a criterion for decompose the HFC because the electrical properties (ion-concentration and mobility) have different characteristics in the intra- and extra-neurite compartments. The MC-SMT uses DWI signals over various diffusion encoding directions for multiple *b*-values. In this paper, we used *b*-values of 0, 800, and 2000 s/mm$^2$ with 0, 16, and 32 gradient directions, respectively. To characterize the microscopic features, MC-SMT uses the spherical mean of signals over multiple diffusion gradient directions for a given *b*-value.

Since the microscopic diffusion features relates with the conductivity, by investigating the diffusion patterns between voxels in the local window, we define a diffusion pattern distance and determine a representative DWI data as a weighted combination of those within the local window. In this paper, we used the diffusion pattern distance, $D(\textbf{r}_c,{\textbf{s}})$, as a norm between two normalized diffusion signals $S_n^t(\textbf{r}_c)$ and $S_n^t({\textbf{s}})$, where $\textbf{r}_c$ is a center voxel of the local window and $S_n^t(\textbf{r}_c)$ denotes a union of total normalized diffusion signals at a voxel $\textbf{r}_c$. The use of estimated IVF knowledge as a partial component of the apparent conductivity is critical to solving the underdetermined problem. Taking into account a presumed isotropic macroscopic voxel and highly heterogeneous electrical charge carriers concentration, we convert the underdetermined equation for a voxel into an over-determined system over a local window of surrounding voxels. In the extra-neurite compartment (intra-neurite compartment), a representative apparent conductivity can be determined as a scalar value over the sliding local window without the strong assumption of the ratio of ion concentrations in the intra- and extra-cellular spaces.

Due to unknown reference values for the extra-neurite conductivity, we analyze the possible error of the reconstructed conductivity by introducing a new indicator function that compares the difference between the conductivity values using the proposed method and those using the assumed ratio of ion concentrations in the intra- and extra-cellular spaces. The indicator function consists of the intrinsic diffusivity, extra-neurite mean diffusivity, IVF, and the referred fixed constant ratio of ion concentrations in the intra- and extra-cellular compartments. Using the estimated indicators, we can specify suspicious regions with errors between the true conductivity and the estimated conductivity due to the use of a fixed ratio of ion concentrations in intra- and extra-cellular compartments.

We propose a non-invasive decomposed MR imaging for the intra-neurite conductivity and extra-neurite conductivity from the HFC :

1. Reconstruct microstructural parameters (intra-neurite volume fraction, intrinsic diffusion coefficient, extra-neurite mean diffusivity) extracted by MC-SMT.
2. Reconstruct the intra-neurite conductivity and extra-neurite conductivity from the HFC.
  Express the HFC as a two compartment model with respect to IVF.
  Change the underdetermined problem at each voxel for the intra- and extra-neurite conductivity into an over-determined problem over a local window of voxels.
  Define a diffusion pattern distance to determine appropriate weights to suppress the noise amplification and sustain the spatial resolution.
  Determine a representative apparent intra- and extra-neurite conductivity by solving the weighted over-determined system.

For rigorous validation and verification of the proposed method, we design a simulation experiment to demonstrate the effect of local window size and diffusion pattern distances. For the simulation study, a two-dimensional modified Shepp-Logan phantom was generated to quantitatively carry out the numerical implementation. Since the proposed method requires a realistic IVF information reflecting microstructural diffusivity properties, we used a real human IVF datum which was recovered using MC-SMT. The accuracy and precision of the reconstructed conductivity distributions were evaluated, and the impact of different noise levels and defect datasets were also investigated. In addition, human experiments were conducted to separate the high-frequency conductivity by analyzing the multi-shell *b*-values DWI data and acquired multi-echo spin echo data. MRI measurements were performed with five healthy volunteers without a documented history of any disease were recruited. Both HFC and IVF were recovered using the measured B1 phase map and the multi-shell DWI data. We suppressed the noise amplification to stably recover the intra- and extra-neurite conductivities by defining a diffusion pattern distance. Furthermore, to verify and test the proposed method for a practical implementation, we conducted a case of cancer that occurs in the brain. We found that the recovered electrical properties provided information on brain conditions differentiated from conventional MR magnitude imaging.

## Results

### Simulation experimental setup

**Figure 1:**
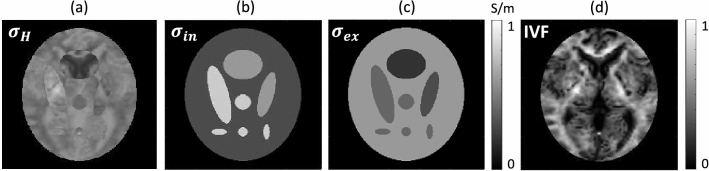
Simulation model. (**a**) High-frequency conductivity. (**b**) Intra-neurite conductivity. (**c**) Extra-neurite conductivity. (**d**) Intra-neurite volume fraction (IVF) recovered from a human brain.

We design a 2-D Shepp-Logan phantom to quantitatively carry out the numerical implementation. Simulations are used to evaluate the decomposition of conductivity over a wider range of noise and resolution. Figure 1b,c denote the simulated noiseless intra-neurite conductivity $\sigma _{in}$ and extra-neurite conductivity $\sigma _{ex}$, respectively. The proposed method requires an intra-neurite volume fraction $\nu _{in}$ to decompose the high-frequency conductivity $\sigma _H$ into $\sigma _H=\nu _{in}\sigma _{in}+(1-\nu _{in})\sigma _{ex}$.

Since the proposed method uses a macroscopic data reflecting microstructural diffusivity properties, for a realistic IVF, we used a recovered human IVF data obtained using MC-SMT. For the realistic IVF, we used DWI data from a healthy 68-year-old male participant with no record of any disease, who was one of a total of 24 cognitive normal participants, used in the paper^17^. The detail of DWI imaging MR acquisitions was same to the following human experimental setup. The IVF in Fig. 1d was estimated by solving the nonlinear equation in Eq. (10) using multi-shell *b*-values of 0, 800, and 2000 s/mm$^2$ (16 and 32 gradient directions for $b=800$ s/mm$^2$ and $b=2000$ s/mm$^2$, respectively).

Figure 1d shows the human IVF image restricted to the support region of Shepp-Logan model. To generate a high-frequency, we first designed the intra-neurite conductivity $\sigma _{in}$ (Fig. 1b) , and extra-neurite conductivity $\sigma _{ex}$ (Fig. 1b). Next, using the equipped IVF, $\sigma _{in}$, and $\sigma _{ex}$, we generated the high-frequency conductivity $\sigma _H$ as a weighted sum of $\sigma _{in}$ and $\sigma _{ex}$. Figure 1a shows the generated high-frequency conductivity $\sigma _H$.

To compare the changes of $L^2$-errors depending on the size of local windows and the diffusion pattern distance, the relative $L^2$-error was defined as

1 $$\begin{aligned} E_{\sigma ^r}=\sqrt{\frac{\sum _{i,j}{\left| \,\sigma ^t(i,j)-\sigma ^r(i,j)\,\right| }^2}{\sum _{i,j}(\sigma ^t(i,j))^2 }} \end{aligned}$$

where $\sigma ^t$ and $\sigma ^r$ denote the noiseless and reconstructed conductivity values in the respective tissue class.

In order to evaluate the performance of the proposed method, it was assumed that the simulated HFC includes Gaussian random white noise with a zero mean and standard deviation. The signal-to-noise ratios (SNR) were 10, 20, and 30 dB with adding different levels of noise.

### Human experimental setup

Five healthy volunteers with no record of any disease were recruited. The participants were positioned inside the bore of a 3T MRI scanner with the head coil in transmit and a 32-channel RF head coil (Ingenia, Philips Medical Systems, the Netherlands). All experimental protocols were approved by the institutional review board of Kyung Hee University Hospital at Gangdong (KHNMC 2020-03-017). All methods were carried out in accordance with the relevant guidelines and regulations and all participants provided written informed consent.

The proposed method applied to a patient case. The prospective study (KHNMC IRB 2019-06-007) was approved by the institutional review board of Kyung Hee University Hospital at Gangdong and informed consent was obtained from the participant patient. The patient (65 years-old, male) had craniotomy and removal of $1.8\times 2.3\times 2.2$ cm$^3$ sized lobulated irregular rim enhancing lesion, suggestive of glioblastoma, at the left occipital lobe at one month before MRI was taken. In addition, the patient had chronic infractions at the bilateral basal ganglia and thalami, microbleeds at the pons and the right occipital lobe, and the moderate small vessel disease.

For MREPT imaging experiments, the multi-spin-echo pulse sequence with multiple refocusing pulses was adopted to minimize the measured noise. Before the data acquisition, we applied a volume shimming method with the volume defined to cover the brain region. Imaging parameters were as follows: repetition time TR = 2800 ms, first echo time TE = 12 ms with 12 ms intervals, number of echoes (NE) = 4, number of excitation (NEX) = 1, slice thickness = 5 mm, number of slices = 20 without a gap between the slices, acquired voxel size = $2 \times 2\times 5$ mm$^3$, reconstructed voxel size = $1\times 1 \times$5 mm$^3$, field-of-view (FOV)= $224\times 224\times 100$ mm$^3$, and SENSE factor = 0, TSE factor = 6, RF shim = “adaptive”, $B_0$ shim = “PB-volume”, slice scan order = interleaved, and regional saturation slab = 45 mm at the feet direction. The total scan time of MREPT sequence was 6 min 19 s.

For the brain tissue segmentation, a sagittal structural three-dimensional (3D) T1-weighted (T1W) image was acquired with the fast field-echo (FFE) sequence, which is similar to the magnetization-prepared rapid acquisition of the gradient echo (MPRAGE) sequence. The imaging parameters were as follows: TR = 9.9 ms, TE = 4.6 ms, FA = 8$^\circ$, and voxel size = $1\times 1 \times 1$ mm$^3$. T2-weighted turbo-spin-echo, fluid-attenuated inversion recovery (FLAIR), and gradient-echo images were also acquired to evaluate any brain abnormalities.

For DWI imaging experiments, the diffusion of water molecules was measured using the single-shot spin-echo echo-planner imaging (SS-SE-EPI) pulse sequence. Diffusion weighting was achieved with two *b*-shells of nominally 800 and 2000 s/mm$^2$ with 16 and 32 gradient directions, respectively. To reduce MR scan time for a practical implementation, we adopt the relatively small number of diffusion gradient directions. Without diffusion weighting, 6 images were also acquired. Imaging parameters were as follows: TR = 15,000 ms, TE = 86 ms, NE = 1, NEX = 1, slice thickness = 2 mm, number of slices = 54 without a gap between the slices, voxel size = $2\times 2 \times 2$ mm$^3$, FOV = $224\times 224\times 108$ mm$^3$, and EPI factor=37, TSE factor = 6, RF shim=“adaptive”, $B_0$ shim = “PB-volume”, slice scan order = interleaved, and regional saturation slab = 45 mm at the feet direction. Total scan times were 2 min 15 s, 4 min 45 s, and 8 min 45 s for *b*-values of 0, 800, and 2000 s/mm$^2$, respectively.

Since the proposed method uses IVF to separate the HFC, we restricted ROI to the white matter (WM) region. The separation of WM tissues using high resolution T1-weighted structural images is used to determine tissue volumes and regions of a given tissue type. To segment WM, Statistical Parametric Mapping version 12 (SPM12) software was used (http://www.fil.ion.ucl.ac.uk/spm/software/spm12/). First, the 3D T1W image and all maps for each volunteer were co-registered. Second, the 3D T1W image was segmented into gray matter, white matter, and cerebrospinal fluid using the computational anatomy toolbox (CAT12) tool (http://www.neuro.uni-jena.de/cat/).

Since the accumulated noise artifacts in the phase signal is inversely proportional to MR magnitude intensity, $\tilde{{\mathscr{S}}}_k,~k=1,3$, the measured phase signal was optimized as a weighted averaging using the weight^25^ of

$$\begin{aligned} w_k = \frac{|\tilde{{\mathscr{S}}}_k|^2}{|\tilde{{\mathscr{S}}}_1|^2+|\tilde{{\mathscr{S}}}_3|^2}, ~k=1,3 \end{aligned}$$.

### Simulation results

**Figure 2:**
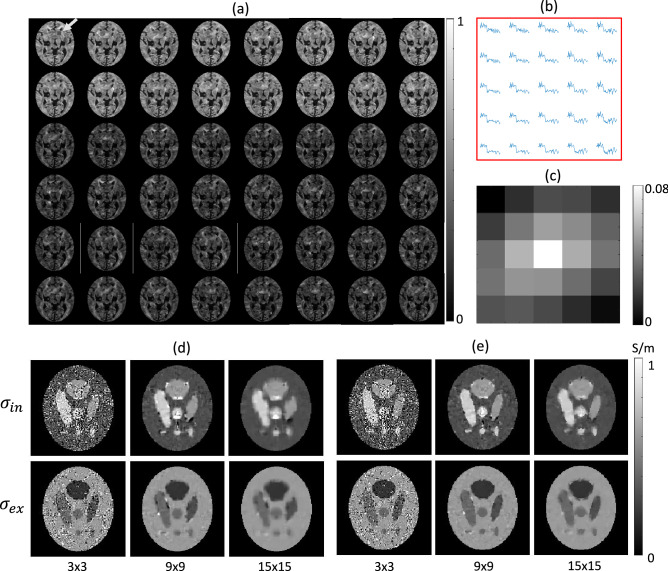
Simulation results. (**a**) Simulated DWI data set. Upper two rows: DWI data corresponding to 16 gradient directions for *b*-value 800 s/mm$^2$. The other rows: DWI data corresponding to 32 gradient directions for *b*-value 2000 s/mm$^2$. (**b**) Simulated DWI signals for each of 25 voxels in the selected window (red blot in (**a**), pointed by the yellow arrow) as a function of *b*-value and diffusion gradient direction (first for 16 different directions with $b = 800$ s/mm$^2$ and then for 32 different directions with $b = 2000$ s/mm$^2$). DWI plot at a voxel depends on the diffusion gradient directions and multi-shell *b*-values of 800 and 2000 s/mm$^2$. (**c**) Determined weighting factors using the diffusion pattern distance in the local window in (**b**). (**d**) Recovered the intra-neurite conductivity, $\sigma _{in}$, and the extra-neurite conductivity, $\sigma _{ex}$, by solving the over-determined system Eq. (18) with evenly distributed weighting factors. Gaussian noise with noise level (20 dB) were added to the high-frequency conductivity. Reconstruct results by changing the window size of $3\times 3$ (first column), $9\times 9$ (second column), and $15\times 15$ (third column). (**e**) Recovered results with weighting factors using the diffusion pattern distance $D(\textbf{r}_c,{\textbf{s}})$ correspond to (**d**).

To generate a diffusion pattern distance map, we modified the human DWI data (6, 16, and 32 directions for *b*-values of 0, 800, and 2000 s/mm$^2$, respectively), which were used to recover the IVF in Fig. 1d. We normalized the DWI data for *b*-values of 800 and 2000 s/mm$^2$ by dividing the average signals of $b=0$. Figure 2a shows the generated DWI data set consisting of a total 48 DWI images. The upper two rows were simulated DWI images corresponding to 16 gradient directions for *b*-value 800 s/mm$^2$ and the other rows were those corresponding to 32 gradient directions for *b*-value 2000 s/mm$^2$. To describe the DWI patterns in a local window, we select a local region with size of $5\times 5$ voxels, the red blot in Fig. 2a, pointed by the yellow arrow.

In the selected local window, Fig. 2b shows the DWI plots at each voxel. The DWI plot at a voxel shows the diffusion signals with respect to the diffusion gradient directions and multi-shell *b*-values of 800 and 2000 s/mm$^2$. Using the generated DWI data, we calculated the diffusion pattern distance, $D(\textbf{r}_c,{\textbf{s}_i})=\frac{{\left\| \,S_n(\textbf{r}_c,:)-S_n({\textbf{s}_i},:)\,\right\| }}{h(\textbf{r}_c)},~i=1,\ldots ,25$, to determine the weighting factors $\omega (\textbf{r}_c,{\textbf{s}}_i),~i=1,\ldots ,25$ in Eq. (19). Here, $S_n(\textbf{r}_c,j),~j=1,\ldots ,48$ denotes the DWI values to estimate IVF. We set the denominator $h(\textbf{r}_c=1)$, which was appropriate for the generated normalized DWI data set.

Figure 2c shows the estimated weighting factors to determine a representative DWI signal at the center voxel in the local window. Figure 2d,e show the recovered the intra-neurite conductivity, $\sigma _{in}$, and the extra-neurite conductivity, $\sigma _{ex}$, by solving the over-determined system Eq. (18). To observe the relation between the noise artifact and diffusion pattern distance depending on the local window size, the high-frequency conductivity was contaminated using additive white Gaussian noise with a noise level of 20 dB. Figure 2d shows the reconstruct results with evenly distributed weighting factors were displayed in by changing the window size of $3\times 3$ (first column), $9\times 9$ (second column), and $15\times 15$ (third column). The evenly distributed weighting factors (${\textbf{W}}$ is the identity matrix) efficiently reduced the noise artifacts of recovered $\sigma _{in}$ (upper row) and $\sigma _{ex}$ (lower row) as moving the window size from $3\times 3$ to $15\times 15$ in Fig. 2d. However, as the window size increases, the evenly distributed weighting factors cause resolution loss because of blur increases with decreasing anomaly size. Using the diffusion pattern distance $D(\textbf{r}_c,{\textbf{s}})$ in Eq. (17), Fig. 2e shows the recovered conductivity images corresponding to those in Fig. 2d. Comparing to the evenly distributed weighting factors, we found that the system Eq. (18) using the weighting factors successfully suppressed the noise artifact while maintaining the resolution of intra- and extra-neurite conductivity.

**Figure 3:**
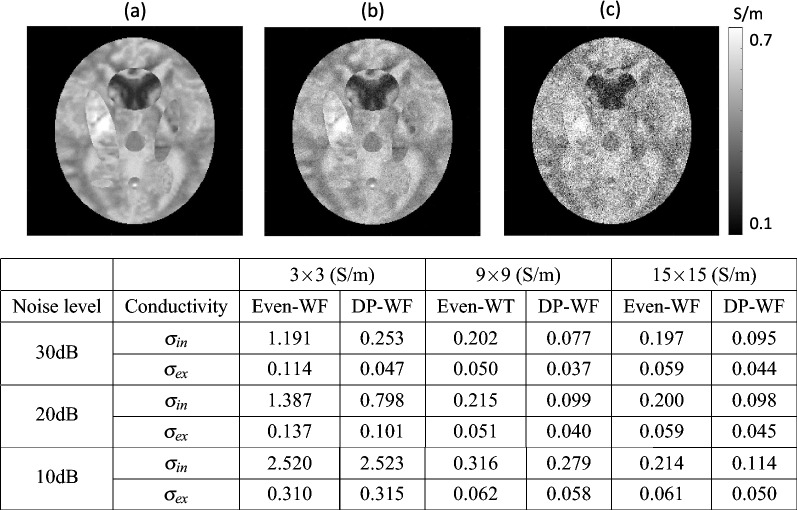
Simulation results. (**a**–**c**) Simulated noise added high-frequency conductivity images from 10 to 30 dB, respectively. The relative $L^2$-error for the reconstructed intra- and extra-neurite conductivity depending on the noise level and local window size. The relative errors were calculated for the evenly distributed weighting factors (Even-WF), and for the weighting factors using the diffusion pattern distance (DP-WF), respectively.

To evaluate the efficiency of diffusion pattern distance depending on the local window size, we evaluated the relative $L^2$-error in Eq. (1). Figure 3a–c show the simulated noise added HFC images, where additive white Gaussian noise was used from 10 dB to 30 dB, respectively. The table in Fig. 3 shows the relative $L^2$-error. The relative $L^2$-error for the reconstructed $\sigma _{in}$ and $\sigma _{ex}$ depended on the noise level, local window size, evenly distributed weighting factors, and evaluated weighting factors using the diffusion pattern distance $D(\textbf{r}_c,{\textbf{s}})$.

### Human experiment results for healthy volunteers

**Figure 4:**
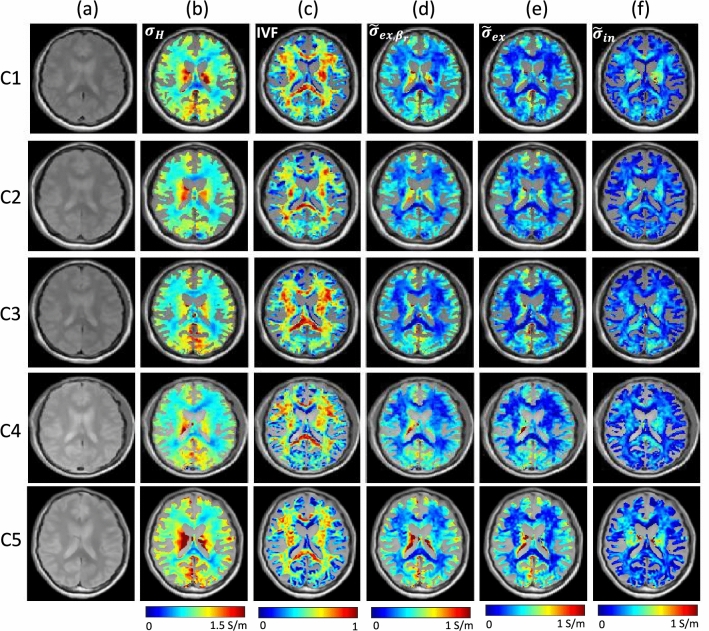
Human experiment results for five healthy volunteers. ROI is selected as the segmented white matter region using SPM. (**a**) Normalized magnitude images. (**b**) Recovered high-frequency conductivity. (**c**) Intra-neurite volume fraction (IVF). (**d**) Recovered apparent extra-neurite conductivity using a reference ratio ($\beta _r=0.41$). (**e**) Recovered apparent extra-neurite conductivity by solving the minimization problem Eq. (18). (**f**) Recovered apparent intra-neurite conductivity using the proposed method.

To quantify the reconstructed intra- and extra-neurite conductivity, $\sigma _{ex}$, and $\sigma _{in}$, from the HFC, $\sigma _H$, by solving the over-determined system Eq. (18), we fixed the local window size as $21\times 21$ and estimated weighting factors using the diffusion pattern distance, $D(\textbf{r}_c,{\textbf{s}})$. Figure 4a shows the spatially normalized magnitude images of the five healthy young volunteers. Figure 4b shows the reconstructed HFC $\sigma _H$ in the WM region segmented by SPM software package. To estimate $\sigma _H$, we used odd echoes of four measured complex to optimize the phase signals. The reconstruction of $\sigma _H$ is achieved by numerical differentiation of measured phase signals. A three-point central difference approximation was used to generate the matrix system ${\textbf{A}}{{\textbf{x}}}={\textbf{b}}$ in Eq. (8) and the diffusion term $c = 0.025$ was added to stabilize the reaction–diffusion equation (7). The conductivity images restricted to the WM region were still influenced by the volume fractions of white matter, gray matter, and cerebrospinal fluid (CSF) in the supported region. The HFC values in WM of the corpus callosum were relatively lower than those in the other regions. By solving the nonlinear model in Eq. (10), the spherical mean signal ${\bar{e}}_b$ in Eq. (9) estimates IVF displayed in Fig. 4c. In Table 1, the mean values of IVF in WM dominant region at the fixed 61-th slice were 0.40492, 0.36758, 0.40992, 0.38716, and 0.36962 for the volunteers (from case 1 to case 5), respectively. The mean between the volunteers was 0.3878 and the standard deviation was 0.0195.

**Table 1:** Human experiment results for five healthy volunteers.

	(a) High-frequency conductivity (S/m)	(b) Intra-neurite volume fraction (IVF)	(c) Apparent extra-neurite conductivity ($\beta _r$ = 0.41) (S/m)	(d) Apparent extra-neurite conductivity (S/m)	(e) Apparent intra-neurite conductivity (S/m)
Case 1	0.40231	0.40492	0.25048	0.20389	0.19859
Case 2	0.36201	0.36758	0.23008	0.19487	0.16775
Case 3	0.37075	0.40992	0.22454	0.19168	0.17925
Case 4	0.37898	0.38716	0.24333	0.20635	0.17283
Case 5	0.44396	0.36962	0.30034	0.25400	0.19065

Mean values of reconstructed results in the segmented white matter region using SPM. Mean values of high-frequency conductivity (a), intra-neurite volume fraction (IVF) (b), recovered apparent extra-neurite conductivity using a reference ratio ($\beta _r=0.41$) (c), recovered apparent extra-neurite conductivity by solving the minimization problem Eq. (18) (d), and recovered apparent intra-neurite conductivity using the proposed method (e).

We subsequently use $\beta _r=0.41$ as the referred constant ratio of ion concentrations in the intra- and extra-cellular compartments^24^ and $\beta$ as the ratio of ion concentrations in the intra- and extra-neurite compartments. Figure 4d shows the apparent extra-neurite conductivity images using the fixed reference ratio $\beta _r=0.41$:

$$\begin{aligned} \tilde{\sigma }_{ex,\beta _r} = \frac{(1-\nu _{in}) \sigma _H}{(1-\nu _{in}) \tilde{\lambda }^{ext} + \nu _{in} \lambda \beta _r}\tilde{\lambda }^{ext} \end{aligned}$$

The perturbed errors in Eq. (14) implies that the estimated apparent extra-neurite conductivity, $\tilde{\sigma }_{ex,\beta _r}$, may contain additional errors in WM focused ROI region, where IVF and the intrinsic diffusion coefficient $\lambda$ are relatively lager than those in the other regions. Within a reasonably perturbed $\beta$, which means that ${\left| \,\beta _\varepsilon \,\right| }$ is relatively small, the indicator $\eta (\tilde{\sigma }_{ex,\beta _r})$ in Eq. (14) denotes the error that may occur between the true conductivity and the estimated conductivity using a reference value $\beta _r$. If $\eta (\tilde{\sigma }_{ex,\beta _r})$ values are large in a region, the recovered conductivity, $\tilde{\sigma }_{ex,\beta _r}$, is suspicious in the region.

Figure 4e shows the reconstructed apparent extra-neurite conductivity $\tilde{\sigma }_{ex} = (1-\nu )\sigma _{ex}$ by solving the minimizing problem Eq. (18) for the local window size $21\times 21$ and weighting factors using the decay pattern map. Compared to the apparent extra-neurite conductivity images, $\tilde{\sigma }_{ex,\beta _r}$, for the reference ratio $\beta _r=0.41$ in Fig. 4d, we found that the recovered $\tilde{\sigma }_{ex}$ in Fig. 4e considerably correlated with $\tilde{\sigma }_{ex,\beta _r}$, but the conductivity values were slightly lower than those for the fixed $\beta _r=0.41$. Figure 4f shows the apparent intra-neurite conductivity image, $\tilde{\sigma }_{in}=\nu _{in}\sigma _{in}$, for the five volunteers.

In Table 1, we calculated the mean values of HFC (a), IVF (b), recovered apparent extra-neurite conductivity using a reference ratio ($\beta _r=0.41$) (c), recovered apparent extra-neurite conductivity by solving the minimization problem Eq. (18) (d), and recovered apparent intra-neurite conductivity using the proposed method (e).

Fig S1 and Fig S2 show the human experiment results for the young five healthy volunteers at the 50-th and 70-th slices, respectively. For the 50-th and 70-th slices, we used same processes for the results at the 61-th slice in Fig. 4. Table S1 and Table S2, we estimated the mean values of HFC, IVF, apparent extra-neurite conductivity using the reference ratio $\beta _r=0.41$, and apparent extra- and intra-neurite conductivity by solving the over-determined system with the local window size $21\times 21$ at the 50-th and 70-th slices, respectively.

### Human experiment results for disease case

To verify the feasibility of the proposed method for a disease case, we separated the apparent intra- and extra-neurite conductivity, $\tilde{\sigma }_{ex}$, and $\tilde{\sigma }_{in}$, using the recovered HFC, $\sigma _H$ with a fixed the local window size as $21\times 21$ and the decay pattern similarity map.

**Figure 5:**
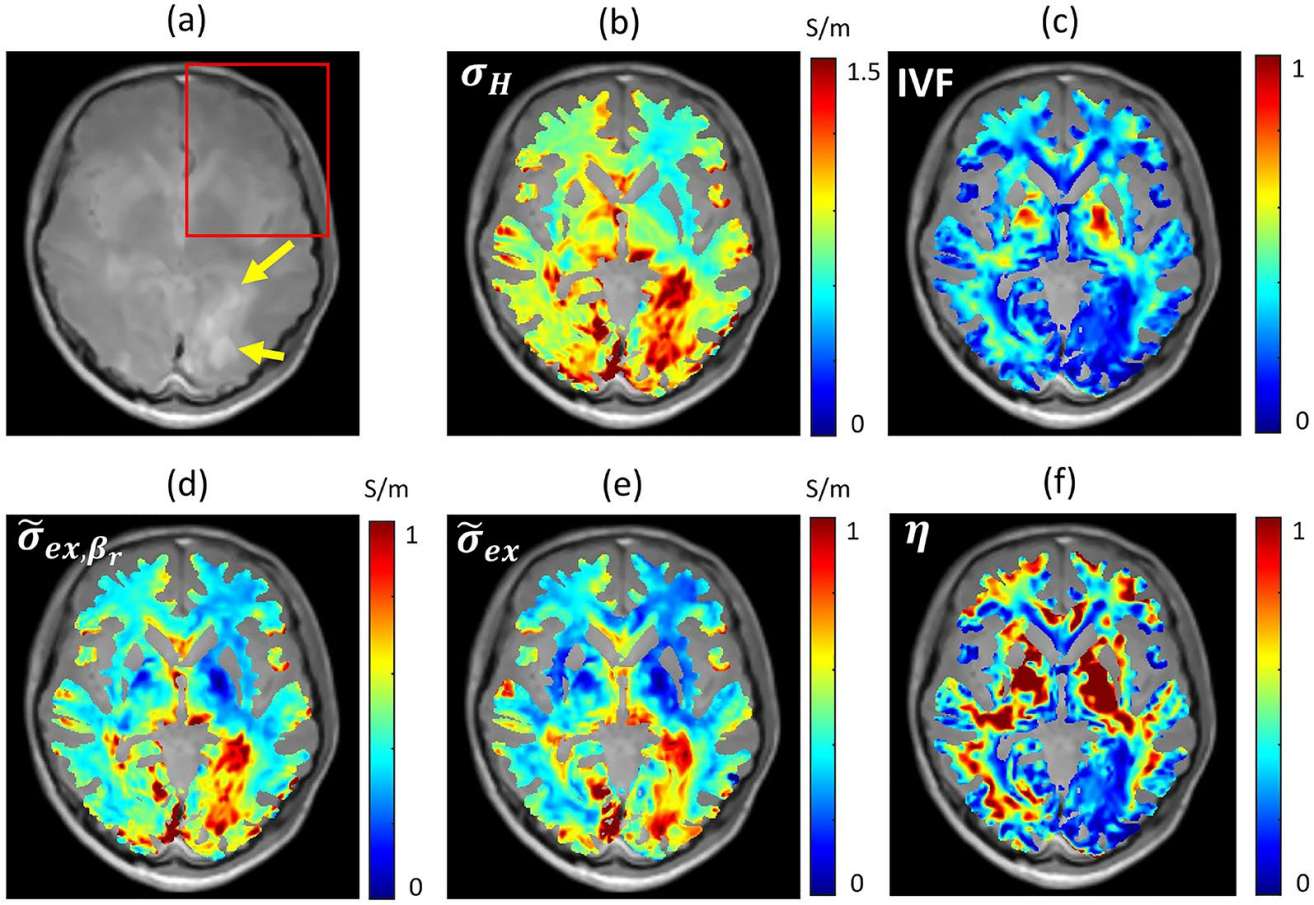
Human experiment results for the case of a patient with brain tumor of the glioblastoma. ROI is selected as the segmented white matter region using SPM. (**a**) Normalized magnitude images. Yellow arrows indicate the lobulated irregular rim enhancing lesion (R1-ROI) and the red rectangle region (R2-ROI) is selected to compare the conductivities. (**b**) Recovered high-frequency conductivity. (**c**) Intra-neurite volume fraction (IVF). (**d**) Recovered apparent extra-neurite conductivity using a reference ratio ($\beta _r=0.41$). (**e**) Recovered apparent extra-neurite conductivity by solving the minimization problem Eq. (18). (**f**) Indicator function $\eta (\tilde{\sigma }_{ex,\beta _r})$ in Eq. (14) from the recovered extra-neurite conductivity using the fixed reference ratio $\beta _r=0.41$.

Figure 5 shows the reconstructed results for the case of a patient with brain tumor of the glioblastoma. We selected a characteristic slice and segmented the white matter region of interest (WM-ROI) using SPM. Figure 5a shows a normalized magnitude image at the 50-th imaging slice. To compare the reconstructed results, the yellow arrows indicate the lobulated irregular rim enhancing lesions. To compare and investigate the reconstructed extra-neurite conductivity, we selected a left region (red rectangle). Figure 5b denotes the recovered HFC by solving the stabilized reaction-diffusion partial differential equation (7). To numerically solve the equation, we adopted the stabilized constant $c=0.03$ in Eq. (7). Figure 5c shows the IVF. For the patient (65-years old, male), the mean value of IVF in R1-ROI was 0.18676, which was lower than the mean value (0.31107) in WM-ROI because of the removal of $1.8\times 2.3\times 2.2$ cm$^3$ sized lobulated irregular rim enhancing lesion. Figure 5d shows the recovered apparent extra-neurite conductivity $\tilde{\sigma }_{ex,\beta _r}$ using the reference ratio $\beta _r=0.41$. The mean value of $\tilde{\sigma }_{ex,\beta _r}$ was 0.70676 S/m in R1-ROI. That was similar to the mean of $\sigma _H$ which was 0.7866 S/m. In R2-ROI, the mean values of $\tilde{\sigma }_{ex,\beta _r}$ and $\sigma _H$ were 0.36963 S/m and 0.48226 S/m, respectively. For the fixed $\beta _r=0.41$, the mean values of indicator function, $\eta (\tilde{\sigma }_{ex,\beta _r})$, were 0.51534, 0,25422, and 0.60880 in WM-ROI, R1-ROI, and R2-ROI, respectively. In Fig. 5f, we displayed the indicator function values in WM-ROI.

Using the indicator function $\eta$, we can estimate the errors between $\tilde{\sigma }_{ex}$ and $\tilde{\sigma }_{ex,\beta _r}$ :

2 $$\begin{aligned} {\left| \,\tilde{\sigma }_{ex}(\textbf{r})-\tilde{\sigma }_{ex,\beta _r}(\textbf{r})\,\right| } \le C{\left| \,\beta _\varepsilon (\textbf{r})\,\right| }\eta (\tilde{\sigma }_{ex,\beta _r}) \end{aligned}$$

Since there is no known method to estimate the ratio of ion concentrations in the intra- and extra-neurite compartments, the quantity $\eta (\tilde{\sigma }_{ex,\beta _r})$ is indirectly related to the error ${\left| \,\tilde{\sigma }_{ex}(\textbf{r})-\tilde{\sigma }_{ex,\beta _r}(\textbf{r})\,\right| }$. If we use the fixed $\beta _r=0.41$ to recover the extra-neurite conductivity, the estimated apparent extra-neurite conductivity $\tilde{\sigma }_{ex,\beta _r}$ is acceptable for a region where the values of $\eta (\tilde{\sigma }_{ex,\beta _r})$ are relatively low by assuming that $\beta _\varepsilon$ is reasonably perturbed for the fixed $\beta _r$. In R1-ROI, the mean value of $\eta (\tilde{\sigma }_{ex,\beta _r})$, 0.25422, was relatively smaller than 0.51534, which was the mean value of $\eta (\tilde{\sigma }_{ex,\beta _r})$ in WM-ROI. In R1-ROI, the mean values of $\tilde{\sigma }_{ex,\beta _r}$ with the fixed $\beta _r=0.41$ and $\tilde{\sigma }_{ex}$ using the proposed method without constraint of $\beta$, were 0.70676 and 0.67329, respectively. Similarly, in R2-ROI, the mean values of $\tilde{\sigma }_{ex,\beta }$ with constrain of $\beta _r$ and $\tilde{\sigma }_{ex}$ without constraint of $\beta$, were 0.36963 and 0.33256, respectively. The mean value of $\eta (\tilde{\sigma }_{ex,\beta _r})$, 0.60880, in R2-ROI was relatively larger than that of $\eta (\tilde{\sigma }_{ex,\beta _r})$, 0.25422, in R1-ROI. For these reasons, the reconstructed $\tilde{\sigma }_{ex,\beta _r}$ with a reference ratio $\beta _r=0.41$ in R2-ROI may contain larger errors compared to those in R1-ROI.

In Table 2, we compared the reconstructed values for the patient case with those for the five volunteers.

**Table 2:** Comparison of the human experiment results for the five volunteers and the patient with brain tumor.

		(a) $\sigma _H$ (S/m)	(b) IVF	(c) $\tilde{\sigma }_{ex, \beta _r}$ (S/m)	(d) $\tilde{\sigma }_{ex}$ (S/m)	(e) $\tilde{\sigma }_{in}$ (S/m)	(f) $\eta (\tilde{\sigma }_{ex,\beta _r})$
Volunteers	WM-ROI	0.39160	0.38784	0.24975	0.21016	0.18181	–
Patient	WM-ROI	0.60088	0.31107	0.47868	0.44993	0.15268	0.51534
	R1-ROI(yellow)	0.78660	0.18676	0.70676	0.67329	0.10352	0.25422
	R2-ROI(red)	0.48226	0.34921	0.36963	0.33256	0.15271	0.60880

Mean values of reconstructed results in WM-ROI, R1-ROI (the region designated by the yellow allows in Fig. 5a), and R2-ROI (the region assigned by the red rectangle in Fig. 5a). (a) Mean values of high-frequency conductivity, (b) intra-neurite volume fraction (IVF), (c) recovered apparent extra-neurite conductivity using a reference ratio ($\beta _r=0.41$), (d) recovered apparent extra-neurite conductivity by solving the minimization problem Eq. (18), (e) recovered apparent intra-neurite conductivity using the proposed method, and (f) indicator function $\eta (\tilde{\sigma }_{ex,\beta _r}):={\frac{\nu _{in}\lambda }{(1-\nu _{in}) \tilde{\lambda }^{ext} +\nu _{in} \lambda \beta _r}}$.

## Discussion

The MREPT techniques are well developed for visualizing the internal electrical conductivity distribution at Larmor frequency by measuring the B1 transceive phase data from MRI. Although a HFC imaging by MREPT provides a whole brain electrical property non-invasively, a low-frequency electrical conductivity has advantages to evaluate information of the intra- and extra-cellular spaces. Transcranial direct current stimulation (tDCS) is a form of neuromodulation that uses constant, the low direct current delivered through electrodes on the head. Recent research on tDCS has shown promising results in various neuroscience research areas^26–28^. However, it is very difficult to directly visualize the internal low-frequency conductivity and/or current density because significantly small amount of injection currents. Moreover, even if the measurements of electrical changes in the brain are allowed in MRI, simultaneously experimented twice within an MRI scanner with/without injection currents are needed to obtain the electrical change by the injection current. In this study, we proposed a method to decompose the recoverable HFC at Larmor frequency without external injection current into the brain.

The MC-SMT model based on the intra- and extra-neurite compartments is considered to investigate the electrical conductivity. MC-SMT requires multi-shell *b*-values, at least three different *b*-values ($b=0$, two positive *b*-values) , to determine the two tissue microstructure parameters, intrinsic diffusivity and IVF. In clinical imaging, apparent diffusion coefficient (ADC) and diffusion tensor (DTI) are commonly calculated with two different *b*-values ($b=0$ and one positive *b*-value). Using a mono-exponential model, the DWI for a fixed *b*-value assumes that all water molecules in a voxel have a single ADC, the ADC, however, also reflects combined diffusion properties of intra- and extra-cellular compartments. Although it is difficult to separate the high-frequency conductivity with the diffusion parameters which follow a mono-exponential model, but it would be meaningful to analyze the relationship between the electrical properties and the combined diffusion coefficients using the mono-exponential model. Multi-shell *b*-values DWIs with multi-diffusion gradient directions have been widely studied to detect multi-diffusion coefficients, related with the microstructure. The MC-SMT model used in this paper does not refer directly to the intra- and extra-cellular spaces, but rather focuses on the ball-and-stick model, which has difficulty in distinguishing diffusion signals from soma or other large cellular domains. Recently, a biophysical model has been proposed for apparent cell body (soma) and neurite density imaging (SANDI) to include the soma size and density in addition to neurite density^29^. However, the SANDI model includes more direction-averaged DWI signal at high *b*-values ($\ge$ 3000 s/mm$^2$) to detect the apparent soma size and density and increases the ill-posedness.

The MC-SMT estimates microstructural features specific to the intra- and extra-neurite compartments in the white matter. A previous study showed that the MC-SMT model stably determines the intra- and extra-neurite volume fraction^30–32^. Since the proposed method depends on the local behavior of IVF, one of the estimated results of MC-SMT, to separate the high-frequency conductivity, we used the estimated IVF as a criterion for decompose $\sigma _H$ because the electrical properties (ion-concentration and mobility) also have different characteristics in the intra- and extra-neurite compartments.

**Figure 6:**
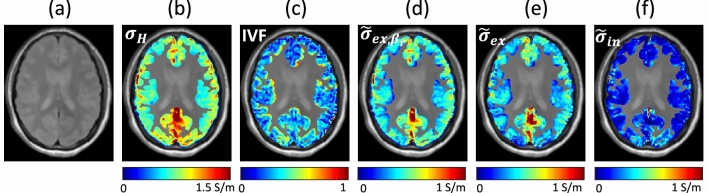
Human experiment results focused on the gray matter. ROI is selected as the segmented gray matter region using SPM. (**a**) Normalized magnitude images. (**b**) Recovered high-frequency conductivity. (**c**) Intra-neurite volume fraction (IVF). (**d**) Recovered apparent extra-neurite conductivity using a reference ratio ($\beta _r=0.41$). (**e**) Recovered apparent extra-neurite conductivity by solving the minimization problem Eq. (18). (**f**) Recovered apparent intra-neurite conductivity using the proposed method.

Due to the property of proposed method relying on a priori information of IVF estimated using MC-SMT, the analysis of electrical conductivity in the white matter region has been focused, but the high-frequency conductivity can be separated in the same way in the gray matter region. Figure 6 shows the reconstructed results in the gray matter region corresponding to the first case (C1) in Fig. 4. The mean conductivity values of $\sigma _H$, $\sigma _{ex, \beta _r}$, and $\sigma _{ex}$ were 0.5134, 0.4209, and 0.3779 S/m, respectively, in the segmented gray matter. Since IVF does not refer to the intra-cellular volume fraction, a rigorous analysis of the separation of high-frequency conductivity and its effect in the gray matter region are required in the future.

As a passive material property, the electrical conductivity of an electrolyte is primarily determined by the concentration and mobility of charge carriers such as ions. The Einstein relation implies that the diffusion coefficient is proportional to the mobility^33^. Extensive researches show that the electrical properties of biological tissues depend on the frequency and type of the tissues^8,34,35^. Under the assumption that the low-frequency conductivity tensor is proportional to the water diffusion tensor, the conductivity tensor imaging techniques without external injected currents have been extensively developed^24,36–40^. In this paper, we only used the information of IVF to separate the HFC. The MC-SMT uses the spherical mean of the signals over multiple diffusion encoding directions for a given *b*-value (two *b*-shells of nominally 800 and 2000 s/mm$^2$ with 16 and 32 gradient directions, respectively, were used in this paper). We plan to investigate the anisotropic electrical conductivity property focused on the restricted and hindered compartments using the estimated microstructural diffusion parameters (IVF, intrinsic diffusion coefficient, extra-neurite mean diffusivity) from MC-SMT and the separated conductivity ($\sigma _{ex}$ and $\sigma _{in}$).

The recently developed methods to decompose the HFC have to assume the unknown ratio, $\beta$, between the intra- and extra-cellular electrical ion concentrations. The strong assumption of $\beta$ is debatable to be accepted as a reliable method. The ratio $\beta$ was estimated using available literature values of intra- and extra-cellular concentrations and hydration diameters of four predominant ions (Na$^+$, Cl$^-$, K$^+$, and Ca$^{2+}$). In this paper, we defined an indicator function, $\eta (\tilde{\sigma }_{ex,\beta _r})$, to analyze the extra-neurite conductivity using the reference constant value $\beta _r=0.41$. Using the characteristics of observable indicator function, $\eta (\tilde{\sigma }_{ex,\beta _r})$, we found a region where the recovered extra-neurite conductivity was reliable. The observation of indicator function is beneficial to determine reliable regions, but using the constant $\beta$ for individual cases is still problematic.

We proposed a new method to separate the HFC at Larmor frequency into the intra- and extra-neurite conductivity without the assumption of constant $\beta$. There are no known reference values for the intra- and extra-neurite conductivity. The proposed method was validated using simulation studies. The simulation study was designed to demonstrate the effect of local window size and diffusion decay pattern and performed to quantitatively carry out the numerical implementation, but more rigorous validation and verification will be worthwhile in future studies. Due to unknown reference values for $\sigma _{ex}$, we tried to indirectly verify the proposed method by comparing the extra-neurite conductivity results with the existing method^23^. In R1-ROI and R2-ROI in Fig. 5a, the behaviors of indicator function $\eta (\tilde{\sigma }_{ex,\beta _r})$ were different (0.25422 in R1-ROI and 0.60880 in R2-ROI). The ratio of conductivity values, $\frac{\tilde{\sigma }_{ex}}{\tilde{\sigma }_{ex,\beta _r}}$, was 0.9526 and 0.8997, respectively, where $\tilde{\sigma }_{ex,\beta _r}$ was the reconstructed apparent extra-neurite conductivity using the fixed $\beta _r$ and $\tilde{\sigma }_{ex}$ was that using the proposed method without constraint of $\beta$. Although the indirect comparisons provide some degree of reliability of the proposed method, it does not directly guarantee the accuracy of separated conductivity values, $\sigma _{ex}$ and $\sigma _{in}$, from the stabilized HFC, $\sigma _H$. In addition to developing methods to prove more rigorous accuracy, it is necessary to prove usefulness through the application of various diseases.

We expect that the recovered intra- and extra-neurite conductivity maps can be applied to the relevant diseases. The separation of high-frequency conductivity can be used for white-matter abnormalities due to processes such as atrophy, changes in the extracellular matrix, and also due to inflammation^41,42^. Since there is no clear explanation for the mechanism, electrical brain stimulation (EBS) studies have relied on computational modeling using reference conductivity values in the brain region. Transcranial direct current stimulation (tDCS), cranial electrotherapy stimulation (CES), electroconvulsive therapy (ECT), and deep brain stimulation (DBS) are promising treatments for human disorders^27,43–45^. Alzheimer’s disease (AD) is the most common neurodegenerative disease. The previous researches have reported that the Na$^+$ in frontal and parietal cortex, and K$^+$ in cerebellum were increased and the diffusivity of water molecule by microstructural disruptions in AD patients was also increased^46,47^. The conductivity imaging may provide clinically useful information for AD diagnosis.

## Conclusion

Based on the fact that conductivity is proportional to the product of mobility and carrier concentration, MC-SMT separates the water molecule movement into the intra- and extra-neurite compartments by analyzing the microstructures and underlying architectural organization of brain tissues. Using the estimated compartmentalized intra-neurite volume fraction, we changed the underdetermined problem to a solvable minimization problem over a local window and determined a representative intra- and extra-neurite conductivity separated from the HFC. To suppress the noise amplification and to sustain a recovered conductivity image without loss of spatial resolution, we used a diffusion pattern distance based on IVF. To quantify the proposed method, we conducted a simulation experiment and human brain experiments (five young healthy volunteers). To verify the reliability of the proposed method, we compared the reconstructed extra-neurite conductivity with the results using previously developed methods. Since the previously developed methods to decompose the HFC have used a strong assumption of a constant ratio of intra- and extra-cellular ion concentrations, we analyzed the possible error of reconstructed conductivity by introducing a new indicator function. Using the indicator function, we indirectly verified the feasibility of the proposed method for a patient with a brain tumor.

## Materials and methods

### Theoretical modeling

#### High-frequency conductivity reconstruction using B1 transceive phase map

Using the applied radio-frequency (RF) excitation pulse, the electrical tissue properties, conductivity $\sigma _H$ and permittivity $\varepsilon _H$, satisfy the time-harmonic Maxwell model:

3 $$\begin{aligned} \nabla ^2\textbf{B}= i \omega \mu _0 \gamma _H \textbf{B}- \frac{\nabla \gamma _H}{\gamma _H} \times (\nabla \times \textbf{B}) \end{aligned}$$

where $\textbf{B}=(B_x,B_y,B_z)$ the magnetic flux density, $\gamma _H=\sigma _H+i \omega \varepsilon _H$ is the admittivity, $\omega$ is the angular frequency, and $\mu _0=4\pi \times 10^{-7}$ N/A$^2$ is the magnetic permittivity of free space^6^.

For the positive (negative) rotating component of the transmit B1 field $B_1^+=\frac{1}{2}(B_x+iB_y)$ ($B_1^-=\frac{1}{2}(B_x+iB_y)$) in Eq. (3), by assuming a local homogeneity ($\nabla \gamma _H=0$), the complex admittivity can be represented as a typical simple algebraic form:

4 $$\begin{aligned} \gamma _H = \frac{\nabla ^2 B^+_1}{i \omega \mu _0 B^+_1} \end{aligned}$$

Without the assumption of local homogeneity, a convection reaction equation-based MREPT formula is expressed as follows^48^:

5 $$\begin{aligned} \psi ^\pm \cdot \nabla \ln (\gamma _H)-\nabla ^2 B_1^\pm +i\omega \mu _0\gamma _H B_1^\pm =0 \end{aligned}$$

where $\psi ^\pm =\left( \frac{\partial B_1^\pm }{\partial x}\mp i\frac{\partial B_1^\pm }{\partial y}+\frac{1}{2}\frac{\partial B_z}{\partial z}, \pm i\frac{\partial B_1^\pm }{\partial x}+ i\frac{\partial B_1^\pm }{\partial y}\pm i\frac{1}{2}\frac{\partial B_z}{\partial z},-\frac{1}{2}\frac{\partial B_z}{\partial x}\mp i\frac{1}{2}\frac{\partial B_z}{\partial y}+\frac{\partial B_1^\pm }{\partial z}\right)$. Using the phasor notation, $B_1^+={\left| \,B_1^+\,\right| }e^{i\phi ^+}$ and $B_1^-={\left| \,B_1^-\,\right| }e^{i\phi ^-}$, by assuming that the ${\left| \,\nabla B_z\,\right| }\approx 0$ and $\sigma _H \gg \omega \varepsilon _H$, the conductivity, related with the imaginary terms in Eq. (5), can be written as a convection-reaction equation as

6 $$\begin{aligned} \nabla \varphi ^{tr}\cdot \nabla \tau _H+\tau _H\nabla ^2\varphi ^{tr}=2\omega \mu _0 \end{aligned}$$

where $\tau _H$ is $\frac{1}{\sigma _H}$ and $\phi ^{tr}=\phi ^++\phi ^-$ denotes the measurable transceive phase.

To stabilize the formula in Eq. (6), an artificial diffusion term is added. In this paer, we use a stabilized phase-based convection reaction equation to estimate the conductivity :

7 $$\begin{aligned} -c\nabla ^2\tau _H+\nabla \varphi ^{tr}\cdot \nabla \tau _H+\tau _H\nabla ^2\varphi ^{tr}=2\omega \mu _0 \end{aligned}$$

where *c* is a scalar value coefficient^48,49^.

Through the numerical approximation, the convection reaction equation (7) leads to the following matrix system using the known boundary information:

8 $$\begin{aligned} {\textbf{A}}{{\textbf{x}}}={\textbf{b}} \end{aligned}$$

where ${\textbf{A}}$ is a staff matrix, ${\textbf{x}}=\left( {\tau _H}_1,\ldots ,{\tau _H}_N \right) ^T$, and ${\textbf{b}}=\left( 2\omega \mu _0,\ldots ,2\omega \mu _0\right) ^T$, respectively. The HFC $\sigma _H$ is recovered by solving Eq. (8), which will be decomposed into the intra- and extra-neurite conductivity.

#### Multi-compartment model for microstructural water molecular diffusivity

To decompose a water diffusion related micro-architecture of biological tissues from macroscopically measured diffusion data, MC-SMT uses the spherical mean signal over the multiple diffusion gradient directions for a specified *b*-value^21^.

For a specified *b*-value, the spherical mean of the diffusion signal over the gradient directions combines the fiber orientation distribution and hence the averaged spherical diffusion signal can be expressed as

9 $$\begin{aligned} {\bar{e}}_b = \int _0^{\pi /2} h_b( \cos \theta ) \sin \theta d \theta \end{aligned}$$

where $h_b$ is the diffusion signal and $\theta$ is the angle between the gradient direction and fiber orientation^22^. In this paper, the diffusion signal was acquired using the single-shot spin-echo echo-planner imaging (SS-SE-EPI) pulse sequence.

The spherical mean signal ${\bar{e}}_b$ in Eq. (9) estimates the effective diffusion coefficients parallel and perpendicular to the axons by solving the following nonlinear model:

10 $$\begin{aligned} {\bar{e}}_b(v_{int}, \lambda ) =\nu _{in} \frac{\sqrt{\pi }\,\,\text {erf} (\sqrt{b \lambda })\,}{2\sqrt{b\lambda }} +(1-\nu _{in} ) \, \text {exp}(-b \lambda _\perp ^{ext}) \frac{\sqrt{\pi }\,\,\text {erf} (\sqrt{b (\lambda -\lambda _\perp ^{ext}) } \,)}{2\sqrt{b(\lambda -\lambda _\perp ^{ext})}} \end{aligned}$$

where

- $\nu _{in}$ : intra-neurite volume fraction (IVF)
- $\lambda \le \lambda _{free}$ : intrinsic diffusion coefficient parallel to the neurites ($\lambda _{free}$ : free-water diffusivity)
- $\lambda ^{ext}_\perp =(1-\nu _{in})\lambda$ : hindered diffusion coefficient modeled as a function of the restricted volume fraction and intrinsic diffusivity
- erf$(x)=\displaystyle \frac{2}{\pi }\int ^x_0 e^{-t^2} dt$ : error function

Using the multiple *b*-values, the ill-posed nonlinear equation (10) can be solved depending on the spherical diffusion signal over multiple gradient directions^21^. The quantified brain microstructure using diffusion MC-SMT estimates the diffusivity related microscopic features specific to the intra- and extra-neurite compartments : the intra-neurite component consists of dendrites and axons that exhibit restricted diffusion, and the extra-neurite compartment refers to the space surrounding the neurites assumed to exhibit hindered diffusion. Due to the Einstein relation, the estimated intra-neurite volume fraction $\nu _{in}$ as a function of diffusivity connects to the electrical conductivity that is proportional to the product of mobility and carrier concentration.

### Decomposition of high-frequency conductivity with respect to intra-neurite volume fraction

The electrical conductivity of biological tissues is primarily determined by the density of charge carriers, their mobility, and the charge electron carries. The conductivity is proportional to the product of mobility and carrier concentration. The recovered HFC $\sigma _H$, at Larmor frequency, is expressed as

11 $$\begin{aligned} \sigma _H=\tilde{\sigma }_{in}+\tilde{\sigma }_{ex}:=\nu _{in}\sigma _{in}+(1-\nu _{in})\sigma _{ex} \end{aligned}$$

where $\nu _{in}$ denotes the volume fraction of the intra-compartment. For simplicity of notation, we write $\tilde{\sigma }_{in}$ and $\tilde{\sigma }_{ex}$ instead of $\nu _{in}\sigma _{in}$ and $(1-\nu _{in})\sigma _{ex}$, respectively.

The decomposed form in Eq. (11) should take into account the followings:

- The decomposed form needs to reflect the electrical properties (mobility and carrier concentration) based on physical electromagnetic phenomena.
- With the reconstructed HFC, each component in Eq. (11) should be recoverable using measurable data for the human brain.

### Relation between intra- and extra-neurite ion concentration

Through the motivation that electrical conductivity is primarily determined by the concentration and mobility of the ion charge carrier, recently various methods have tried to recover relatively low-frequency dominant conductivity in the extra-cellular (or extra-neurite) compartment by relying on a pre-defined biological compartment model^23,24,50^. The previously proposed ideas show that the electrical properties of biological tissues depending on electrical frequency characteristics can be separated from the high-frequency conductivity without dc currents injection.

By representing ${\overline{c}}_i$ and ${\overline{c}}_e$ as the apparent intra- and extra-neurite ion concentration, the apparent intra-neurite ion concentration can be expressed as

12 $$\begin{aligned} {\overline{c}}_i = \beta {\overline{c}}_e \end{aligned}$$

Using $\beta$ in Eq. (12) and the Einstein relation, the extra-neurite conductivity is represented as

13 $$\begin{aligned} \tilde{\sigma }_{ex} =\tilde{\sigma }_{ex,\beta } = \frac{(1-\nu _{in}) \sigma _H}{(1-\nu _{in}) \tilde{\lambda }^{ext} + \nu _{in} \lambda \beta }\tilde{\lambda }^{ext} \end{aligned}$$

where $\nu _{in}$ and $\lambda$ are estimated by solving the Eq. (10) and $\tilde{\lambda }^{ext}$ is the extra-neurite mean diffusivity, which is defined as $\tilde{\lambda }^{ext} = (1- \frac{2}{3}\nu _{in})\lambda$^2,23,24^.

However, in spite of the possibility of separation of the HFC, a fundamental problem is that there is no available way to estimate the ratio $\beta$ in Eq. (12)^24^. For the human brains, a reference ratio $\beta _r=0.41$ has been used by adopting the reference values of the intra- and extra-cellular ion concentrations of four predominant ions including Na$^+$, Cl$^-$, K$^+$, and Ca$^{2+}$^2,24^.

To investigate the error when using the reference value $\beta _r=0.41$, we estimate the apparent extra-neurite conductivity $\tilde{\sigma }_{ex}$ as the perturbed concentration ratio $\beta =\beta _r+\beta _\varepsilon$:

14 $$\begin{aligned} \tilde{\sigma }_{ex}&=\frac{(1-\nu _{in}) \sigma _H \tilde{\lambda }^{ext}}{(1-\nu _{in}) \tilde{\lambda }^{ext} + \nu _{in} \lambda \beta _r} \frac{1}{1+\frac{\nu _{in} \lambda \beta _\varepsilon }{(1-\nu _{in})\tilde{\lambda }^{ext} +\nu _{in} \lambda \beta _r }} \nonumber \\&= \tilde{\sigma }_{ex,\beta _r} +{\mathscr{O}}\left( {\frac{\nu _{in} \lambda \beta _\varepsilon }{(1-\nu _{in})\tilde{\lambda }^{ext} +\nu _{in}\lambda \beta _r }}\right) \nonumber \\&= \tilde{\sigma }_{ex,\beta _r} +{\mathscr{O}}\left( \beta _\varepsilon \eta (\tilde{\sigma }_{ex,\beta _r})\right) \end{aligned}$$

where $\tilde{\sigma }_{ex,\beta _r}$ denotes the apparent extra-neurite conductivity using the fixed reference ratio $\beta _r=0.41$ and $\eta (\tilde{\sigma }_{ex,\beta _r}):={\frac{\nu _{in}\lambda }{(1-\nu _{in}) \tilde{\lambda }^{ext} +\nu _{in} \lambda \beta _r}}$ is an indicator function for the error $\tilde{\sigma }_{ex}-\tilde{\sigma }_{ex,\beta _r}$. Here, the ratio $\frac{{\mathscr{O}}(\varepsilon ) }{\varepsilon }$ stays bounded as $\varepsilon \rightarrow 0$.

Considering that the actual $\beta$ is unknown, by assuming a relatively small $\beta _\varepsilon$, the indicator $\eta (\tilde{\sigma }_{ex,\beta _r})$ in Eq. (14) is a factor of the error that may occur between the true conductivity and the estimated conductivity due to the use of fixed $\beta _r=0.41$.

### Representative apparent extra-neurite conductivity

Diffusion weighted MRI reflects a complicated structural information through the water mobility in a biological tissue. From the Einstein relation, the mobility of a charge carrier is proportional to the diffusivity, where the proportional constant is related to the Bolzman constant and the absolute temperature. Assuming that the mobility of charge carriers is proportional to the mobility of water molecules when they are present in the same microscopic environment, the recovered IVF is deeply related to the mobility of charge carriers.

Solving the underdetermined problem in Eq. (11) depends on the appropriate use of a priori IVF information as a partial component of the apparent conductivity. Taking into account a presumed isotropic macroscopic voxel, inside highly heterogeneous electrical charge carriers concentration, we change the underdetermined equation for a voxel into an over-determined problem over a local window of voxels. Due to the complexity of IVF, for a relatively small window ${\text{ w }}(\textbf{r}_c)=\{\textbf{r}_1,\ldots , \textbf{r}_m\}$ surrounding a given voxel $\textbf{r}_c$, it is possible to determine a representative conductivity values:

15 $$\begin{aligned} \min _{\sigma _{in}(\textbf{r}_c), \, \sigma _{ex}(\textbf{r}_c)}\Vert \sigma _H(\textbf{r}_i)-\big (\nu _{in}(\textbf{r}_i)\sigma _{in}(\textbf{r}_c)+(1-\nu _{in}(\textbf{r}_i))\sigma _{ex}(\textbf{r}_c)\big ) \Vert \end{aligned}$$

A representative conductivity, $\sigma _{in}(\textbf{r}_c)$ and $\sigma _{ex}(\textbf{r}_C)$ in the intra- and extra-neurite compartment, can be determined as a scalar value over a sliding window ${\text{ w }}(\textbf{r}_c)$, instead of assigning conductivity values on a voxel by voxel basis. The over-determined problem Eq. (15) can be formulated as a linear over-determined system over a sliding window ${\text{ w }}(\textbf{r}_c)$ :

16 $$\begin{aligned} {\textbf{A}}{\textbf{x}}(\textbf{r}_c)={\textbf{b}} \end{aligned}$$

where

$$\begin{aligned} {\textbf{A}}=\left[ \begin{array}{cc} \nu _{in}(\textbf{r}_1) &{} (1-\nu _{in}(\textbf{r}_1)) \\ \vdots &{}\vdots \\ \nu _{in}(\textbf{r}_m) &{} (1-\nu _{in}(\textbf{r}_m)) \end{array} \right] ,\quad \\ {\textbf{x}}(\textbf{r}_c)=\left[ \begin{array}{c} \sigma _{in}(\textbf{r}_c) \\ \sigma _{ex}(\textbf{r}_c) \end{array} \right] , \quad \text{ and }\quad {\textbf{b}}=\left[ \begin{array}{c} \sigma _H(\textbf{r}_1) \\ \vdots \\ \sigma _H(\textbf{r}_m) \end{array} \right] . \end{aligned}$$

The proposed method extracts the electrical information of the intra- and extra-neurite compartments in relatively low resolution conductivity by changing the underdetermined problem to a solvable over-determined system via IVF.

### Diffusion pattern similarity weight

For a given voxel $\textbf{r}$, in an imaging region, MC-SMT typically measures DWI data for multi-shell *b*-values to estimate anisotropic diffusion properties in the each compartment. For each *b*-value $b_k$ ($k=1,\ldots , N_b$), let ${\mathscr{D}}^{b_k}=\{\textbf{g}_1,\ldots ,\textbf{g}_{M_k}\}$ be the diffusion gradient (DG) directions at a *b*-value $b_k$, $S^{b_k}_i$, $i=1,\ldots , M_k$, be the measured diffusion MR signals for the DG directions in ${\mathscr{D}}^{b_k}$. We denote $S^t_n(\textbf{r})$ as a union of total normalized diffusion signals $S^t_n(\textbf{r},:)=\bigcup _{k=1}^{N_b}\bigcup _{i=1}^{M_k} \frac{S^{b_k}_i (\textbf{r})}{S^0(\textbf{r})}$, where $S^0(\textbf{r})$ is the MR signal without applying diffusion gradient.

To suppress the noise amplification and to estimate the feasible conductivity values, $\sigma _{in}(\textbf{r}_c)$ and $\sigma _{ex}(\textbf{r}_c)$, we define a diffusion pattern distance:

17 $$\begin{aligned} D(\textbf{r}_c,{\textbf{s}}):=\frac{{\left\| \,S_n^t(\textbf{r}_c)-S_n^t({\textbf{s}})\,\right\| }}{h(\textbf{r}_c)} \end{aligned}$$

Using a diagonal weight matrix ${\textbf{W}}$, the over-determined optimization problem Eq. (15) becomes

18 $$\begin{aligned} \min _{{\textbf{x}(\textbf{r}_c)}=(\sigma _{in}(\textbf{r}_c),\sigma _{ex}(\textbf{r}_c))}{\left\| \,{\textbf{W}\left( Ax(\textbf{r}_c)- b\right) }\,\right\| } \end{aligned}$$

To determine the diagonal weight matrix ${\textbf{W}}$, the non-spatial diffusion pattern distance $D(\textbf{r}_c,{\textbf{s}})$ provides a weighting factor $\omega$:

19 $$\begin{aligned} \omega (\textbf{r}_c,{\textbf{s}}):= \frac{1}{\zeta _{\textbf{r}_c}}\exp (-D(\textbf{r}_c,{\textbf{s}})) \end{aligned}$$

where $\zeta _{\textbf{r}_c}:=\sum _{{\textbf{s}}\in {\text{ w }}(\textbf{r}_c)} \exp (-D(\textbf{r}_c,{\textbf{s}}))$ is a normalization constant such as $\sum _{{\textbf{s}}\in {\text{ w }}(\textbf{r}_c)}\omega (\textbf{r}_c,{\textbf{s}})=1$. The parameter $h(\textbf{r}_c)$ quantifies the diffusion pattern depending on multi-shell *b*-values. The filtering parameter $h(\textbf{r}_c)$ is selected by taking into account the noise variance of measured images $S_n^t$.

## Supplementary Information

Supplementary Information.

## Data Availability

The datasets used and/or analysed during the current study available from the corresponding author on reasonable request.
