# Supplementary material for: High frequency conductivity decomposition by solving physically constraint underdetermined inverse problem in human brain
Source: Sci Rep. 2023 Feb 25;13:3273. doi: 10.1038/s41598-023-30344-1 (PMC9968322; doi:10.1038/s41598-023-30344-1)
Supplement: Supplementary file 1 — Supplementary Information. [file 41598_2023_30344_MOESM1_ESM.pdf]

Supplementary Material for "High Frequency Conductivity Decomposition by Solving Physically Constraint Underdetermined Inverse Problem in Human Brain"

Oh-In Kwon[1,*], Mun Bae Lee[1], and Geon-Ho Jahng[2,*]

[1]Department of Mathematics, College of Basic Science, Konkuk University, Seoul, 05029, Korea
[2]Department of Radiology, Kyung Hee University Hospital at Gangdong, College of Medicine, Kyung Hee University, Seoul, 05278, Korea

Corresponding author : *Oh-In Kwon (oikwon@konkuk.ac.kr)
Corresponding author : *Geon-Ho Jahng (ghjahng@gmail.com)

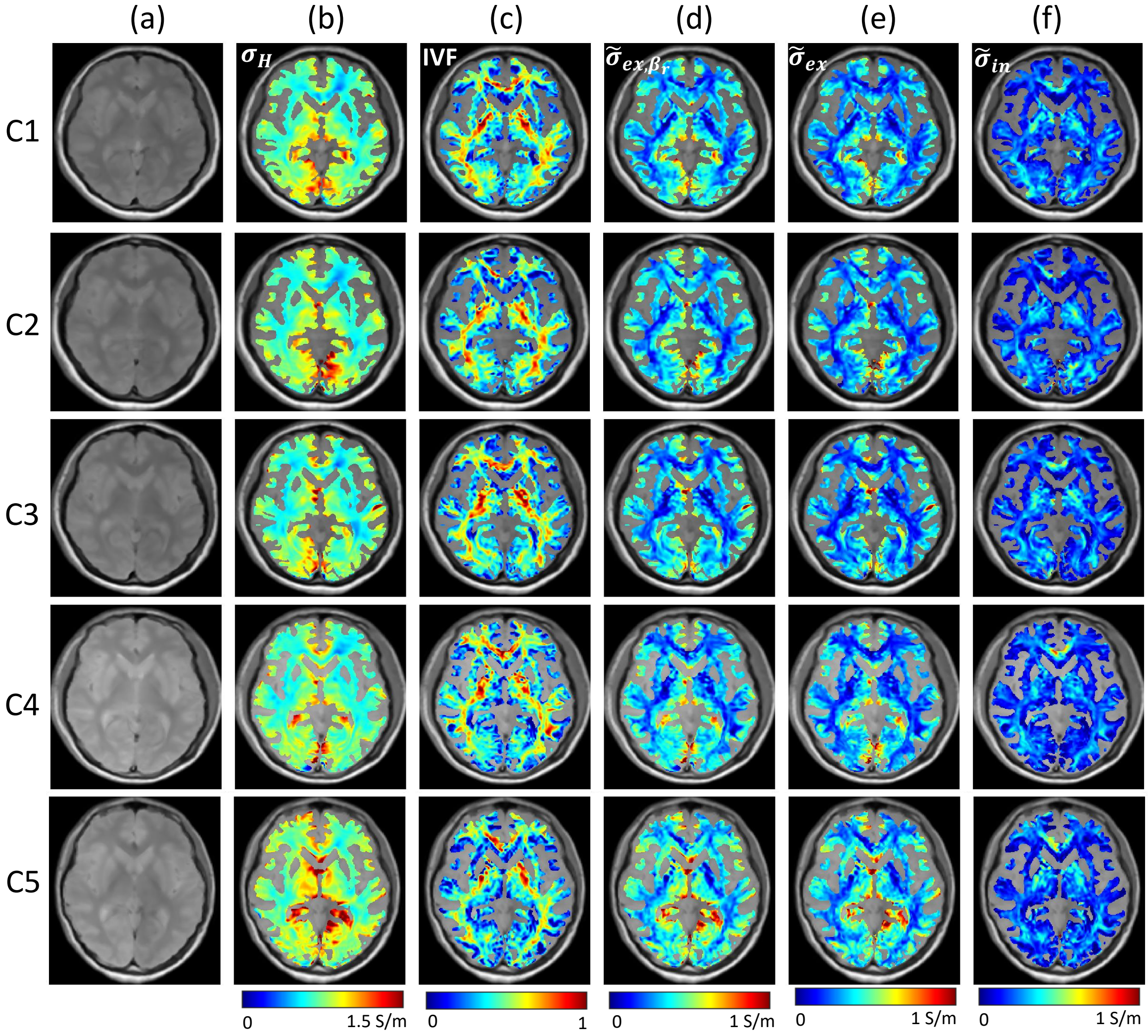

Supplementary figure 1: Human experiment results for five healthy volunteers. Imaging slice is the 50-th slice. ROI is selected as the segmented white matter region using SPM. (a) Normalized magnitude images. (b) Recovered high frequency conductivity. (c) Intra-neurite volume fraction (IVF). (d) Recovered apparent extra-neurite conductivity using a reference ratio ($\beta_r = 0.41$). (e) Recovered apparent extra-neurite conductivity using the proposed method. (f) Recovered apparent intra-neurite conductivity using the proposed method.

	(a) High-frequency conductivity (S/m)	(b) Intra-neurite volume fraction (IVF)	(c) Apparent extra-neurite conductivity ($\beta_r = 0.41$) (S/m)	(d) Apparent extra-neurite conductivity (S/m)	(e) Apparent intra-neurite conductivity (S/m)
Case 1	0.49478	0.44535	0.32625	0.28469	0.21084
Case 2	0.49003	0.43713	0.32528	0.28365	0.20734
Case 3	0.47774	0.44941	0.31298	0.27123	0.20752
Case 4	0.47726	0.43120	0.32108	0.28243	0.19582
Case 5	0.54872	0.38099	0.39600	0.35290	0.19706

Supplementary table 1: Human experiment results for five healthy volunteers. Imaging slice is the 50-th slice. Mean values of reconstructed results in the segmented white matter region using SPM. Mean values of high-frequency conductivity (a), intra-neurite volume fraction (IVF) (b), recovered apparent extra-neurite conductivity using a reference ratio ($\beta_r = 0.41$) (c), recovered apparent extra-neurite conductivityusing the proposed method (d), and recovered apparent intra-neurite conductivity using the proposed method (e).

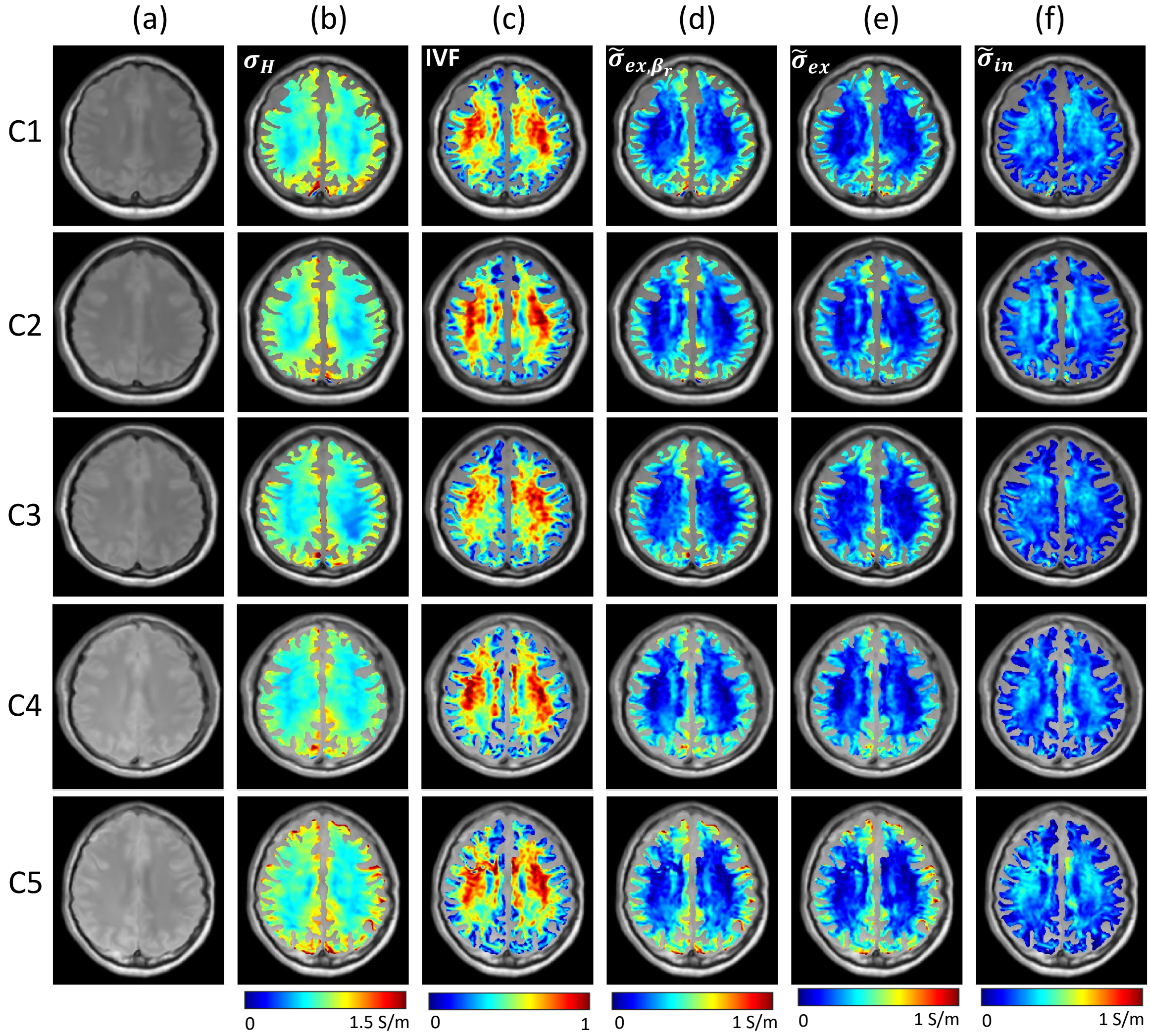

Supplementary figure 2: Human experiment results for five healthy volunteers. Imaging slice is the 70-th slice. ROI is selected as the segmented white matter region using SPM. (a) Normalized magnitude images. (b) Recovered high-frequency conductivity. (c) Intra-neurite volume fraction (IVF). (d) Recovered apparent extra-neurite conductivity using a reference ratio ($\beta_r = 0.41$). (e) Recovered apparent extra-neurite conductivity using the proposed method. (f) Recovered apparent intra-neurite conductivity using the proposed method.

	(a) High-frequency conductivity (S/m)	(b) Intra-neurite volume fraction (IVF)	(c) Apparent extra-neurite conductivity ($\beta_r = 0.41$) (S/m)	(d) Apparent extra-neurite conductivity (S/m)	(e) Apparent intra-neurite conductivity (S/m)
Case 1	0.46152	0.53887	0.26256	0.22982	0.23234
Case 2	0.45499	0.53480	0.25884	0.22269	0.23235
Case 3	0.43464	0.52042	0.25792	0.23035	0.20471
Case 4	0.44703	0.53994	0.25165	0.22277	0.22431
Case 5	0.48427	0.51493	0.28680	0.25719	0.22815

Supplementary table 2: Human experiment results for five healthy volunteers. Imaging slice is the 70-th slice. Mean values of reconstructed results in the segmented white matter region using SPM. Mean values of high-frequency conductivity (a), intra-neurite volume fraction (IVF) (b), recovered apparent extra-neurite conductivity using a reference ratio ($\beta_r = 0.41$) (c), recovered apparent extra-neurite conductivityusing the proposed method (d), and recovered apparent intra-neurite conductivity using the proposed method (e).
